# Liquid dynamics sloshing in cylindrical containers: A 3D free-surface reconstruction dataset

**DOI:** 10.1016/j.dib.2020.106546

**Published:** 2020-11-28

**Authors:** Alessia Simonini, Donato Fontanarosa, Maria Grazia De Giorgi, Maria Rosaria Vetrano

**Affiliations:** a EA department, von Karman Institute for Fluid Dynamics, Waterloosesteenweg 72, Sint-Genesius-Rode, Belgium; b TIPs Laboratory, Université Libre de Bruxelles, CP 165/67 50 av. F.D. Roosvelt, Bruxelles 1050, Belgium; c Department of Engineering for Innovation, University of Salento, Piazza Tancredi 7, Lecce, Italy; d Department of Mechanical Engineering, TME KU Leuven, Celestijnenlaan 300A, Heverlee B-3001, Belgium

**Keywords:** Sloshing, 3D reconstruction, Reference image topography, RIT, Free Surface, Damping

## Abstract

The present dataset provides experimental measurements of the 3D liquid/gas interface shape during lateral water sloshing in a partially filled cylindrical container. The measurement technique used to acquire the data is the Reference Image Topography [1] based on a Synthetic Schlieren Free-Surface Reconstruction method [2]. A modified version of the processing algorithm has been used. This one transforms the coordinate system from Cartesian to polar so that the computational domain only includes the area where the fluid is present. Moreover, it uses the conservation of the fluid volume into the investigated area that permits to obtain the absolute height. This allows overcoming the strong limitation of the RIT method regarding the inability to detect changes of the mean surface height, at the condition that the complete and only liquid domain recorded in the images is used in the inversion algorithm. The complete details of the post-processing of the images is reported in the paper associated to this DIB [3]. The dataset includes the external excitation history and maps of the liquid/gas interface acquired during the experiment. These data are considered fundamental for the validation of CFD simulations of sloshing and of simplified theoretical models. A set of 12 test cases are reported in this DIB. A part of these test cases refers to steady state sloshing and a part to sloshing damping. In the last cases, also the 3D map of damping coefficients, calculated using the logarithmic decrement method, is provided.

**Specifications Table**

**Table:** 

Subject	Aerospace Engineering
Specific subject area	Experimental fluid science, two-phase flows, liquid sloshing, Free-Surface Synthetic Schlieren, Particle Image Velocimetry, Reference Image Topography, free-surface dynamics
Type of data	Maps Tables Tensors
How data were acquired	The hardware used to acquire the dataset is composed of a sloshing table, a cylindrical container, an Optical Displacement Sensor (ODS) unit with conditioner (1 µm accuracy), a signal generator, a NI DAQ system and a Flow Master Stereo-PIV system (LaVision). The PIV system consists of a double cavity Nd:Yag pulsed laser (λ=532 nm, *E* = 125 mJ, pulse duration = 9 ns), and two double frame cameras (2360 × 1776 pixels) equipped with a Nikkor objective (35 mm f/2.8). The laser pulse emission has been set at the maximum rate (i.e., 30 Hz). A Program Time Unit (PTU) synchronizes the cameras exposure time and the laser pulse emission. The commercial PIV code, DaVis 8 (La Vision), is used both for PIV data acquisition and processing. Finally, an external trigger signal, generated by a Stanford Signal Generator, allows the synchronization of the acquired images and the ODS data. A Matlab post-processing code, developed by the authors in the framework of a confidential research project, transforms the PIV results in instantaneous maps of the liquid/gas interface displacements.
Data format	Raw data: Matlab data file .mat, Table data .csv Secondary data: images .png
Parameters for data collection	Data refer to measurements of the liquid/gas interface shape during sloshing inside a cylindrical container partially filled with water, and laterally forced by means of sinusoidal excitation with amplitude of 1.2 mm. The water was at room temperature and atmospheric pressure, i.e. 27.5 °C and 100,313 Pa respectively. The radius of the container was *R* = 40 mm while the non-dimensional liquid level height h/R was set to 1.5 and 2. The free surface reconstruction has been performed for several values of the frequency ratio *K_Ω_* which is defined as the ratio between the forcing frequency and the first natural frequency, i.e. *K_Ω_ = ω_f_/ω_11_*. The data were acquired at 30 Hz, meaning that 30 maps over 1 second of experiment are available for each test case.
Description of data collection	Particle Image Velocimetry has been performed in order to retrieve an optical displacement field related to the shape of the liquid/gas interface. Using methodology developed in the Free-Surface Synthetic Schlieren, the optical displacement field is converted into a free surface height field. The external excitation signal for the two dataset, steady state and damping is obtained using the Optical Displacement Sensor (ODS).
Data source location	Von Karman Institute for Fluid Dynamics Waterloosesteenweg 72 Sint-Genesius-Rode Belgium
Data accessibility	With the article
Related research article	A. Simonini, D. Fontanarosa, M.G. De Giorgi, M.R. Vetrano, Mode characterization and damping measurement of liquid sloshing in cylindrical containers by means of Reference Image Topography, Experimental Thermal and Fluid Science, Volume 120, 2021, 110232, ISSN 0894-1777, https://doi.org/10.1016/j.expthermflusci.2020.110232.

## Value of the Data

• The datasets included in this manuscript provide a clear description of the physical phenomena taking place at the liquid/gas interface during sloshing in a partially filled cylindrical container.
• Steady state numerical simulations of sloshing phenomena, need to be validated using experimental data sets for which an accurate description of the initial and boundary conditions.
• The accuracy of theoretical models of sloshing damping, often based on simplified assumption, can be compared to experiments to evaluate the impact of the used simplifications/assumptions.

## 1 Data Description

The presented data refer to liquid sloshing inside a cylindrical container with internal diameter of 40 mm. The container is partially filled with water, and laterally forced by means of sinusoidal excitation having an amplitude *X_0_* = 1.2 mm. The water was at room temperature and atmospheric pressure, i.e. 27.5 °C and 100,313 Pa respectively. The presented data set correspond to the test matrix presented in Table 1. They have been obtained by varying both the level to container radius ratio, *h/R*, and the frequency ratio *K_Ω_* which is defined as the ratio between the forcing frequency and the first natural frequency, i.e. *K_Ω_ =* ω_f_/ω_11_. The data were acquired at a frequency of 30 Hz.

**Table 1:** Experimental test matrix. For all test cases *X*_*0*_=1.2 mm and the data acquisition frequency is *f* = 30 Hz. RIT data: *N*_*x*_ and *N*_*y*_ are the spatial dimensions along x and y axes, respectively; *N*_*t*_ is the temporal dimension; *Δs* is the grid step in mm; *Δt* is the time step in s.

Test Case	h/R	*K_Ω_*	Regime	RIT data					Raw Data Files
				*N_x_*	*N_y_*	*N_t_*	*Δs* [mm]	*Δt* [s]	
1	1.5	0.79	*Steady*	*134*	*134*	*100*	*0.59*	*0.03*	*Test#_steady.mat* *Test#.csv*
2	1.5	0.9		*134*	*134*	*100*	*0.59*	*0.03*	
3	1.5	1.1		*134*	*134*	*100*	*0.59*	*0.03*	
4	2	0.8		*142*	*142*	*100*	*0.56*	*0.03*	
5	2	0.9		*142*	*142*	*100*	*0.56*	*0.03*	
6	2	1.1		*142*	*142*	*100*	*0.56*	*0.03*	
7	1.5	0.89	*Transient*	*134*	*134*	*300*	*0.59*	*0.03*	*Test#_damped.mat* *Test#.csv* *Test#_dampingMap.png* *Test#_rsquare_dampingMap.png*
8	1.5	1.1		*135*	*135*	*350*	*0.59*	*0.03*	
9	2	0.9		*142*	*142*	*300*	*0.56*	*0.03*	
10	2	1.1		*142*	*142*	*350*	*0.56*	*0.03*	
11	2	0.9		*133*	*133*	*300*	*0.59*	*0.03*	
12	2	1.1		*133*	*133*	*500*	*0.60*	*0.03*	

The data set is divided in two parts. Test cases going from 1 to 6 refers to steady state sloshing, achieved by continuously forcing the container at constant frequency. The second part of data have been acquired during a transient phase obtained by braking the container and leaving the liquid freely damping.

The instantaneous maps of the liquid/gas interface are provided together with the external excitation signal as raw data, for the test cases reported in Table 1. Please note that the symbol “#” is used here to denote a specific test case number.

1 Files named **test#_steady.mat**. Data Format: Matlab workspace data. The files contain the dataset corresponding to the liquid/gas interface height of the liquid during sloshing in steady state. They are reconstructed by means of RIT and expressed as three-dimensional double-precision data type tensor ±6% accuracy. In particular, it composed of three variables:
  a *x-coordinate*: 2D double-precision data type tensor, size [*N_x_, N_t_*]. Each column of the tensor represents the x-coordinate space of the image (units: millimeter) made of *N_x_* spatial steps at a specified time point. The number of time points is *N_t_*, sampled at 30 Hz frequency. The x-coordinate is along the excitation direction.
  b *y-coordinate*: 2D double-precision data type tensor, size [*N_y_, N_t_*]. Each column of the tensor represents the y-coordinate space of the image (units: millimeter) made of *N_y_* spatial steps at a specified time point. The number of time points is *N_t_*, sampled at 30 Hz frequency.
  c *surfaceHeight*: 3D double-precision data type tensor, size [*N_x_, N_y_, N_t_*]. The first two dimensions define the map of the reconstructed free surface height (units: millimeter; ±6% accuracy) into the image space drawn by *x-coordinate* and *y-coordinate* subspaces, at a specified time point. Instead, the number of time points (the third tensor dimension) is *N_t_*, sampled at 30 Hz frequency.
2 Files named **test#_steady.csv**. Data Format: comma separated value table. The files contain the displacement of the sloshing table along the excitation axis (x-coordinate) as a function time, measured by means of an Optical Displacement Sensor (ODS). Data are presented as tables composed by two columns, named *timeODS* and *displacementODS*, reporting time (units: second) and the sloshing table displacement (units: millimeter) respectively. the table displacement was measured with an accuracy of 18 µm and a resolution of 1 µm. The reading provided by the sensor has been truncated to 3 decimal places in accordance with the ODS resolution of 1 μm.
3 Files named **test#_damped.mat**. Data Format: Matlab workspace data. The files contain the dataset corresponding to the liquid/gas interface height of the liquid during sloshing in transient regime (damping of the fluid after the braking of the table). They are reconstructed by means of RIT and expressed as three-dimensional double-precision data type tensor ±6% accuracy. In particular, it composed of three variables:
  a *x-coordinate*: 2D double-precision data type tensor, size [*N_x_, N_t_*]. Each column of the tensor represents the x-coordinate space of the image (units: millimeter) made of *N_x_* spatial steps at a specified time point. . The number of time points is *N_t_*, sampled at 30 Hz frequency. The x- axis corresponds to the excitation axis.
  b *y-coordinate*: 2D double-precision data type tensor, size [*N_y_, N_t_*]. Each column of the tensor represents the y-coordinate space of the image (units: millimeter) made of *N_y_* spatial steps at a specified time point. The number of time points is *N_t_*, sampled at 30 Hz frequency.
  c *surfaceHeight*: 3D double-precision data type tensor, size [*N_x_, N_y_, N_t_*]. The first two dimensions define the map of the reconstructed free surface height (units: millimeter; ±6% accuracy) into the image space drawn by *x-coordinate* and *y-coordinate* subspaces, at a specified time point. Instead, the number of time points (the third tensor dimension) is *N_t_*, sampled at 30 Hz frequency.
4 Files named **test#_dampingMap.png**. Data Format: png figure. The files consist in a figure presenting the local slosh damping δ (units: 1/s), which has been computed using the logarithmic decrement method [3] on the whole surface. The resolution of the .png data file is 600 dpi.
5 Files named **test#_rsquare_dampingMap.png.** Data Format: png figure. The files consists in a figure presenting the coefficient of determination *R^2^* (units: dimensionless) of the logarithmic decrement computation as a map of the goodness-of-fit measure. This last one is inversely proportional to the percent measurement error defined as $(1-R^{2})\cdot 100$ [%]. The resolution of the .png data file is 600 dpi.

The damping (δ) maps and the *R^2^* damping maps do not show data that present an *R^2^* value below 0.5. This corresponds mainly to the regions located in correspondence of the nodal line of the mode. In fact, the nodal position corresponds to the position with lower expected and observed displacements. Smaller displacements, which are almost zero in general, are associated to very high errors.

## 2 Experimental Design, Materials and Methods

The reported datasets result from the use of a novel optical non-intrusive technique, named Reference Image Topography (RIT). This technique is a combination of the Free-Surface Synthetic Schlieren (FS-SS) and the Particle Image Velocimetry (PIV) [1,2].

The RIT technique is based on the evaluation of the apparent deformation of a pattern when this one is observed through a deformed gas/liquid interface. This deformation is then translated into variation of liquid/gas interface height using a numerical integration procedure. For the test case presented here, the pattern is constituted by small fluorescent particles mixed to the liquid and illuminated by a laser sheet.

**Fig. 1:**
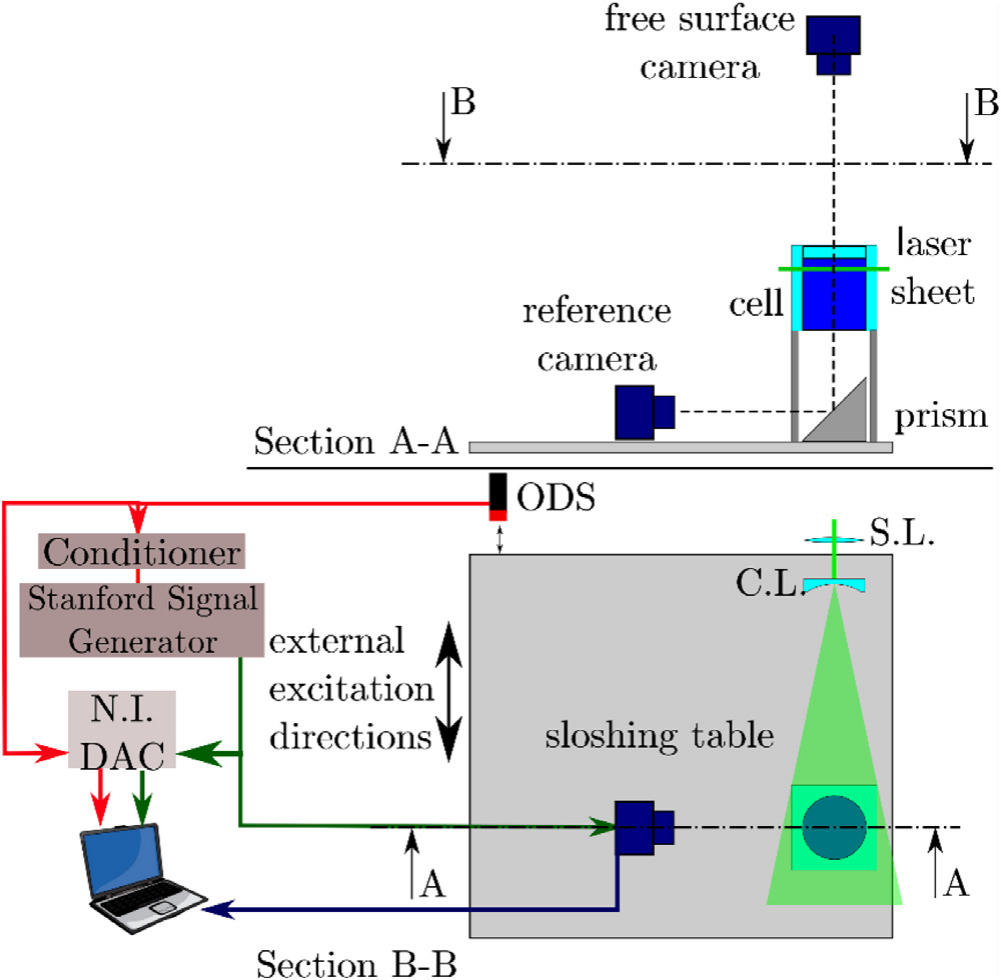
Sketch of the experimental apparatus [3].

The hardware used to acquire this dataset is composed of a sloshing table, a cylindrical container, an Optical Displacement Sensor (ODS) unit with conditioner (1 µm accuracy, 18 μm accuracy), a signal generator, a NI-DAQ (National Instrument-Data Acquisition) system and a PIV system model LaVision Flow Master Stereo-PIV. The PIV system consists of a double cavity Nd:Yag pulsed laser (λ=532 nm, *E* = 125 mJ, pulse duration = 9 ns), and two double frame cameras (2360 × 1776 pixels) equipped with a Nikkor objective (35 mm f/2.8). The laser pulse emission has been set at the maximum laser pulse rate, i.e. 30 Hz. As far as the synchronization of the camera exposure and the laser pulse emission is concerned, this is performed via a Program Time Unit (PTU). Fig. 1 shows the schematic of the whole experimental apparatus under operation: the laser beam emitted by the laser head is firstly focused by a spherical lens and then spread by a cylindrical lens in a laser sheet perpendicular to the tank axis.

Water is seeded using hydrophobic fluorescent red particles (Cospheric). These ones are made hydrophilic by properly mixing with the *Tween 80* biocompatible surfactant and deionized water. The choice of the particle concentration has been based on preliminary PIV test, by verifying a uniform particle distribution and a stable mixture homogeneity. Surface tension measurements performed in the framework of similar experiments have shown that the particles do not have any measurable impact on the liquid contact angle and surface tension. The properties of the particles are summarized in Table 2.

**Table 2:** Properties of fluorescent Cospheric particles.

Diameter [μm]	Density [kg *m* ^−^ ^3^]	Absorption band [nm]	Emission peak [nm]
10–45	998	460–6505	605

The commercial code DaVis 8 (LaVision) [4] is used for PIV data acquisition and processing. Finally, an external trigger signal, generated by the Stanford Signal Generator, allows the synchronization of the acquired images (sampling frequency: 30 Hz) and the ODS data (sampling frequency: 200 Hz).

Table 3 reports the processing parameters used for the image cross-correlation. The instantaneous displacement vector fields are finally used as input for the developed RIT processing algorithm.

**Table 3:** Parameter used to obtain the displacement vector field. WS: windows size, WOR: windows overlap, SNR: signal to noise ratio.

Initial WS [px2]	Final WS [px2]	WOR [%]	SNR limit [-]
128 × 128	48 × 48	75	1.8

The spatial calibration of the images was of 0.05 mm/px.

A Matlab post-processing code, developed by the authors in the framework of a confidential research project, transforms the PIV results in instantaneous maps of the liquid/gas interface height (±6% accuracy). For the test cases of the transient regime (test 7 to test 13), by using the logarithmic decrement method, the local slosh damping has been retrieved based on the temporal evolution of the reconstructed free surface height.

The free-surface reconstruction algorithm is based on the following Matlab functions:

• *intgrad2, which calculates* the inversion of the gradient operator using second order centered differences [5],
• *surfheight* provided in PIVMat [6], which computes the surface height in a rectangular domain starting from the optical displacement, the pattern-free surface distance, the pattern-top camera distance, the refractive index of the liquid and the optical center coordinates.

**Fig. 2:**
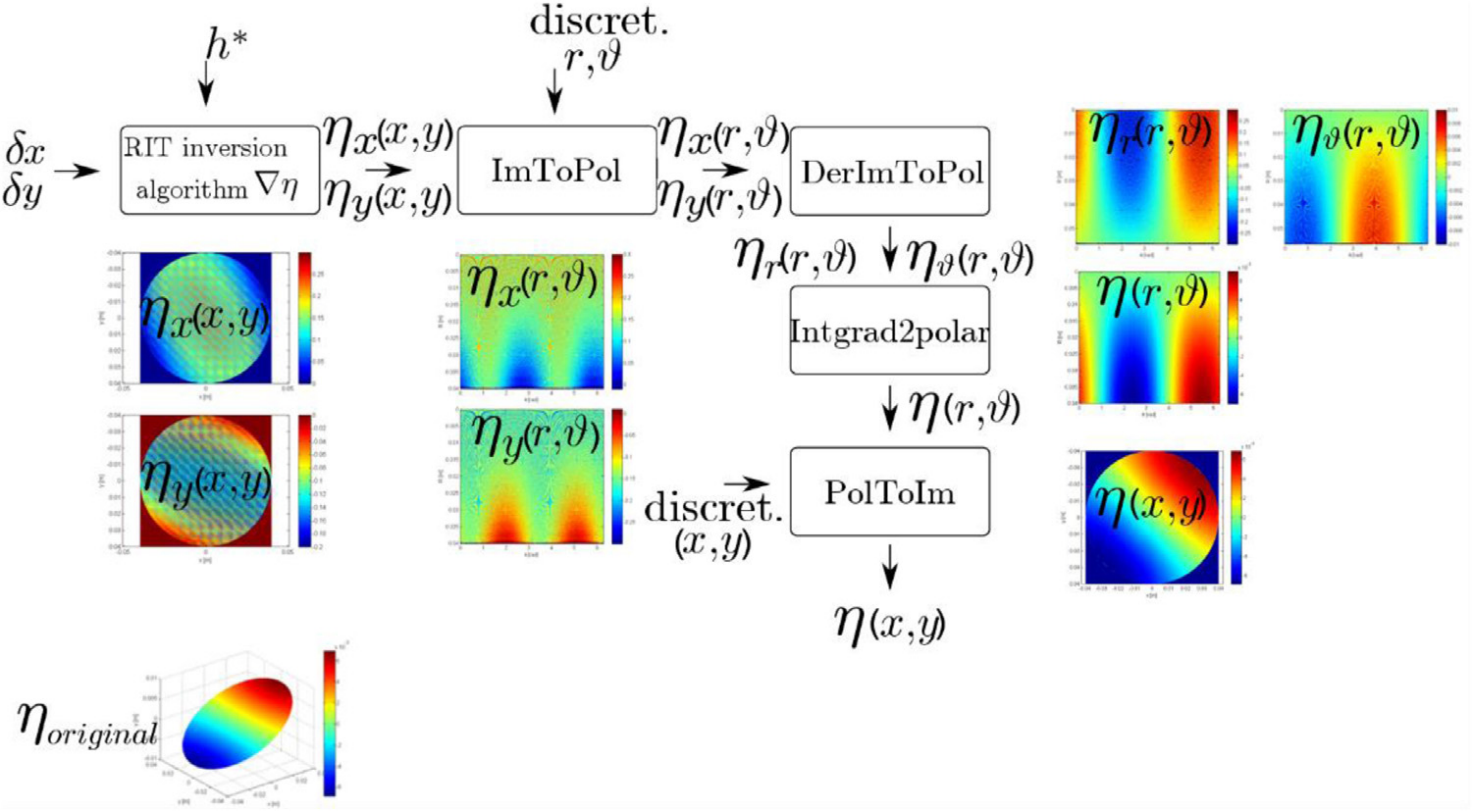
Flowchart of the RIT post-processing methodology for liquid/gas interface reconstruction [3].

Using the aforementioned Matlab functions, the inversion algorithm was able to operate with circular domains. Fig. 2 shows the fundamental blocks of the inversion algorithm:

1 the function *ImToPol*, which transforms the original Cartesian reference system of the physical domain of the image to a polar coordinate system;
2 the function *Pol2Im*, which consists in the inverse transformation of *ImToPol*;
3 the function *DerImToPol*, which transforms the derivative from a Cartesian reference system to a polar one.

The center of the circular domain must be precisely specified at the beginning of the transformation, and the discretization size in the radial and circumferential directions must be assigned. It is worth underlining that this transformation concerns only the reference system: the Cartesian gradient $\Delta \eta \left(x,y\right)=\left(\eta_{x}\left(x,y\right),\eta_{y}\left(x,y\right)\right)$ is transformed into the polar gradient$\;\Delta \eta \left(\theta ,r\right)=\left(\eta_{x}\left(\theta ,r\right),\eta_{y}\left(\theta ,r\right)\right)$ as follows:

(1) $$\eta_{r}=\eta_{x}\cos\left(\theta \right)+\;\eta_{y}\text{sin}\left(\theta \right)$$

(2) $$\eta_{\theta }=\left(-\eta_{x}\sin\left(\theta \right)+\;\eta_{y}\text{cos}\left(\theta \right)\right)R$$

where *η* is the local free surface displacements, (*r, θ*) denote the polar coordinates, (*x,y*) denote the Cartesian coordinates, and *R* is the tank radius. Consequently, the gradient operator can be written with linear algebra:

(3) $$G_{r}H\left(r,\theta \right)=\eta_{x}\left(r,\vartheta \right)$$

(4) $$G_{\theta }H\left(r,\theta \right)=\eta_{\theta }\left(r,\vartheta \right)$$

where *G_r_* and *G_θ_* are two sparse matrices of size (*n_r_, n_θ_*) defining the linear combination of elements of H to produce each gradient. *G_r_* is defined producing second order centered differences, with the treatment of the borders as forward and backward; *G_θ_* is defined, producing second-ordered differences everywhere and considering the continuity of the tangential direction. Instead, *n_r_* and *n_θ_* are the number of step points along the radial and circumferential directions, respectively. Consequently, they are treated as follows:

(5) $$f_{1}^{\prime }=\frac{f_{i,2}-\;f_{i,n_{\theta }}}{\Delta \theta_{i,n_{\theta }}+\;\Delta \theta_{i,1}}$$

(6) $$f_{n_{\theta }}^{\prime }=\frac{f_{i,1}-\;f_{i,n_{\theta }-1}}{\Delta \theta_{i,n_{\theta }-1}+\;\Delta \theta_{i,n_{\theta }}}$$

Concerning the calculation of the absolute height, thanks to the conservation of the volume of sloshing, the following expression of the absolute free surface height at the generic point A related to the reconstructed free surface height *h_s_* is obtained

(7) $$\eta_{A}\left(x,y\right)=h_{S}\left(x,y\right)+K$$

with *K* being the correction factor. *K* can be estimated as a function of the initial level of fluid *h_i_* and the average reconstructed free surface height ${\bar{h}}_{S}$, as reported in Eq. (8).

(8) $$K=h_{i}-{\bar{h}}_{S}$$

The authors refer to [3] for the further details concerning the physical interpretation of the experimental results and a more complete explanation of the basic optical considerations related to the RIT technique.

## Ethics Statement

All authors declare that:

1) this material has not been published in whole or in part elsewhere;
2) the manuscript is not currently being considered for publication in another journal;
3) all authors have been personally and actively involved in substantive work leading to the manuscript and will hold themselves jointly and individually responsible for its content.

## Declaration of Competing Interest

The authors declare that they have no known competing financial interests or personal relationships that have, or could be perceived to have, influenced the work reported in this article.

## Appendix Supplementary materials

Image, application 1
