# Supplementary figures and images for: Liquid dynamics sloshing in cylindrical containers: A 3D free-surface reconstruction dataset
Source: Data Brief. 2020 Nov 28;33:106546. doi: 10.1016/j.dib.2020.106546 (PMC7725740; doi:10.1016/j.dib.2020.106546)

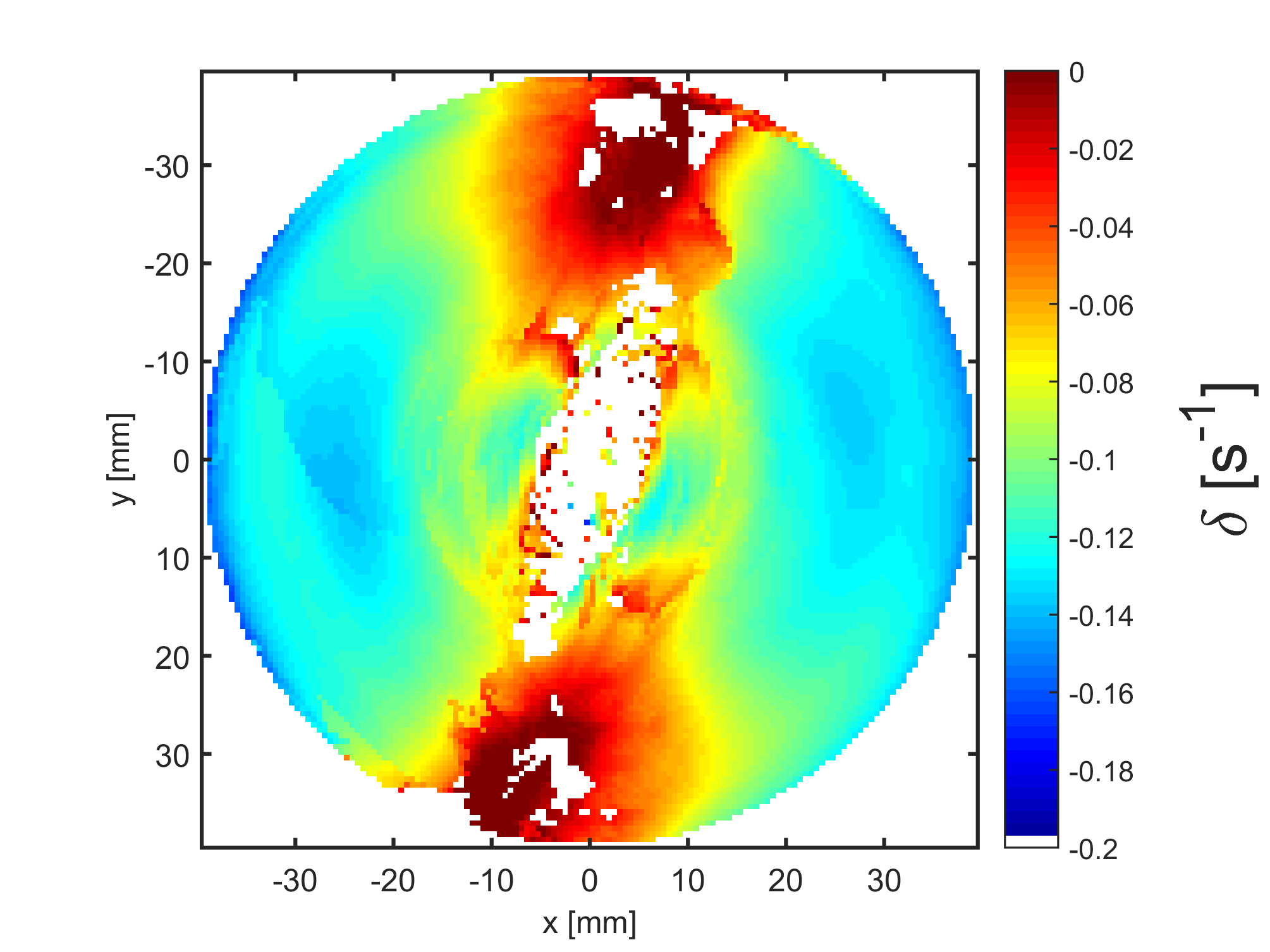

Supplement: Supplementary file 1 [file mmc1.zip › dibData_new/test10_dampingMap.png]

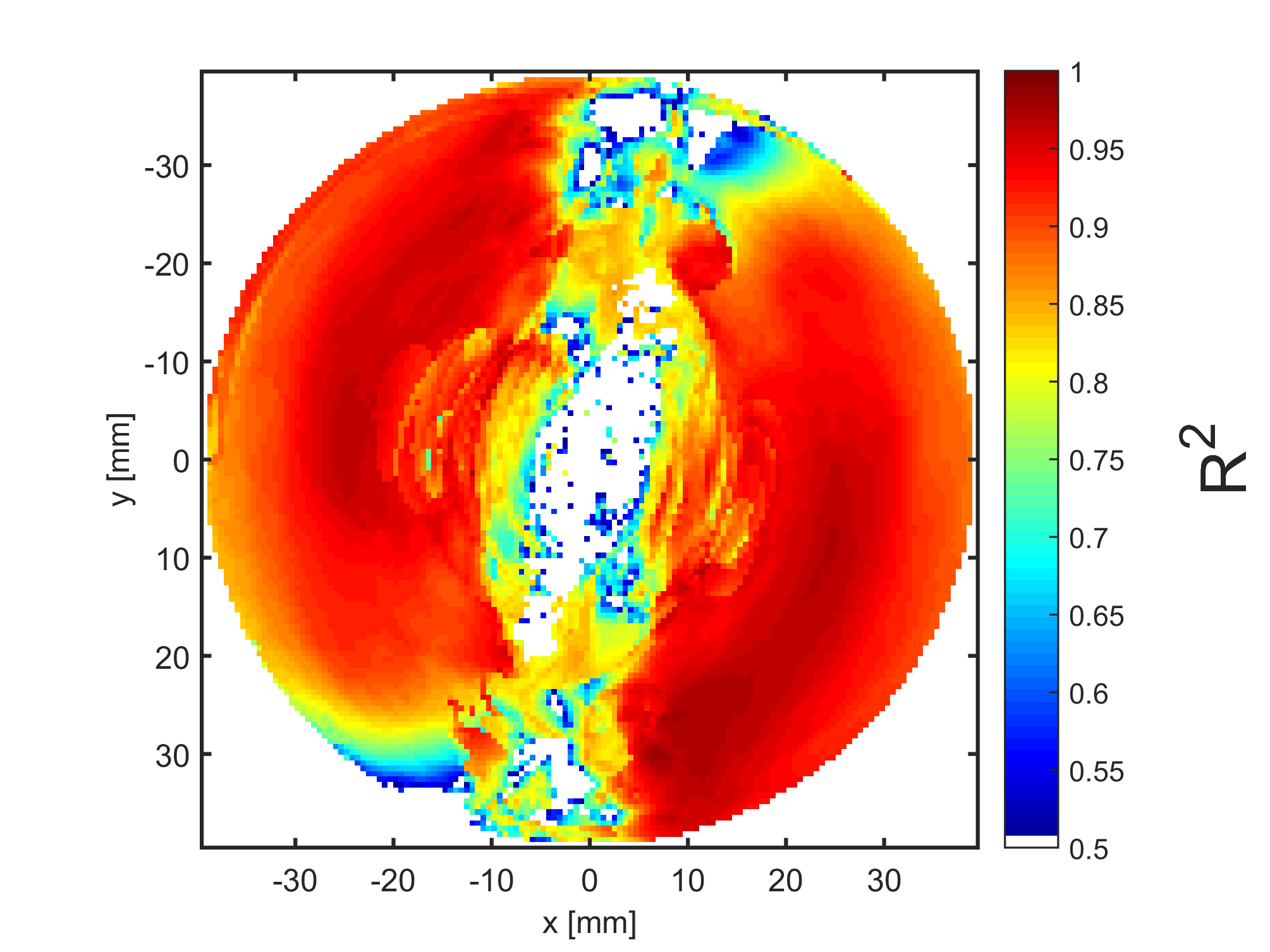

Supplement: Supplementary file 1 [file mmc1.zip › dibData_new/test10_rsquare_dampingMap.png]

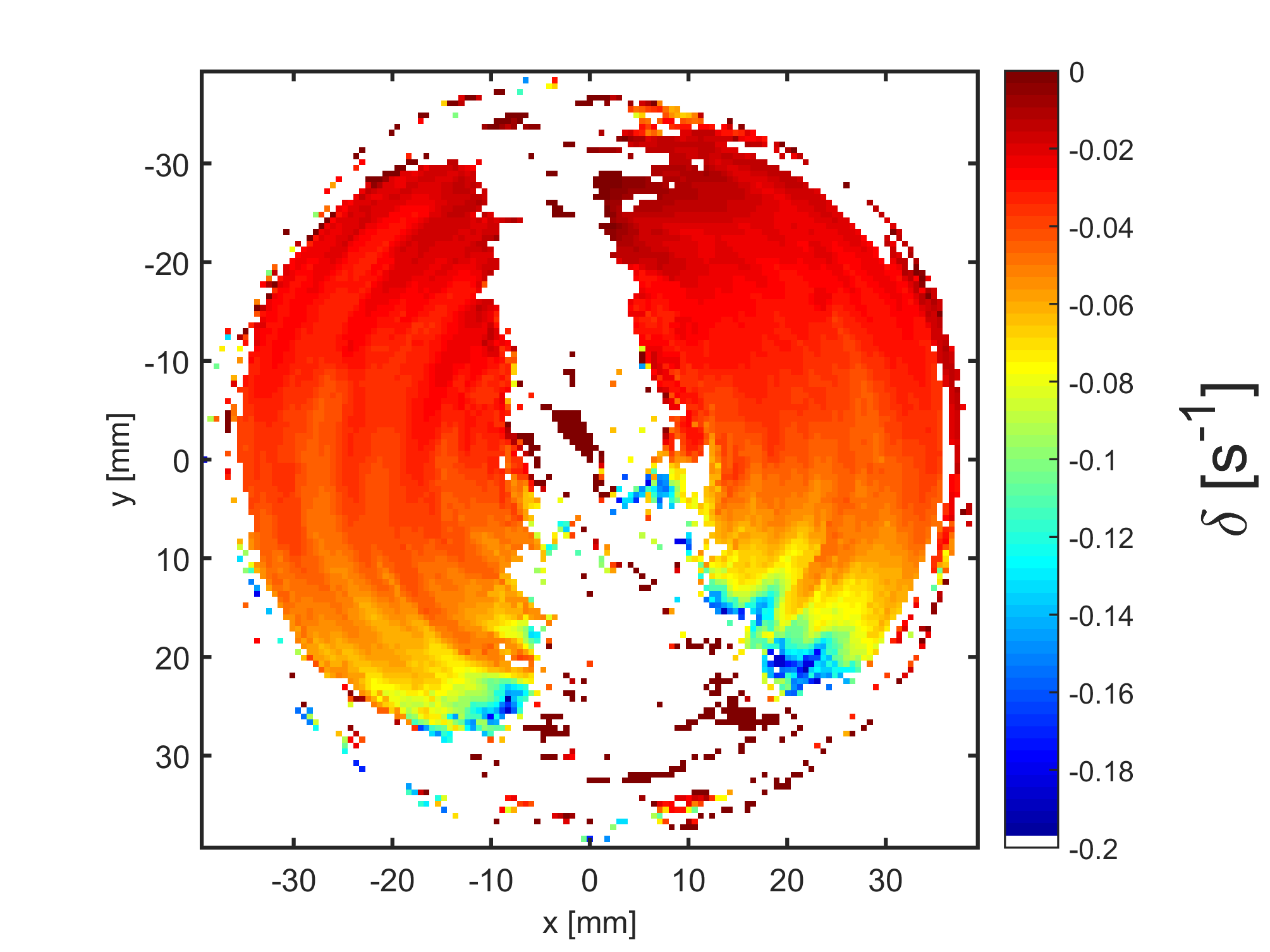

Supplement: Supplementary file 1 [file mmc1.zip › dibData_new/test11_dampingMap.png]

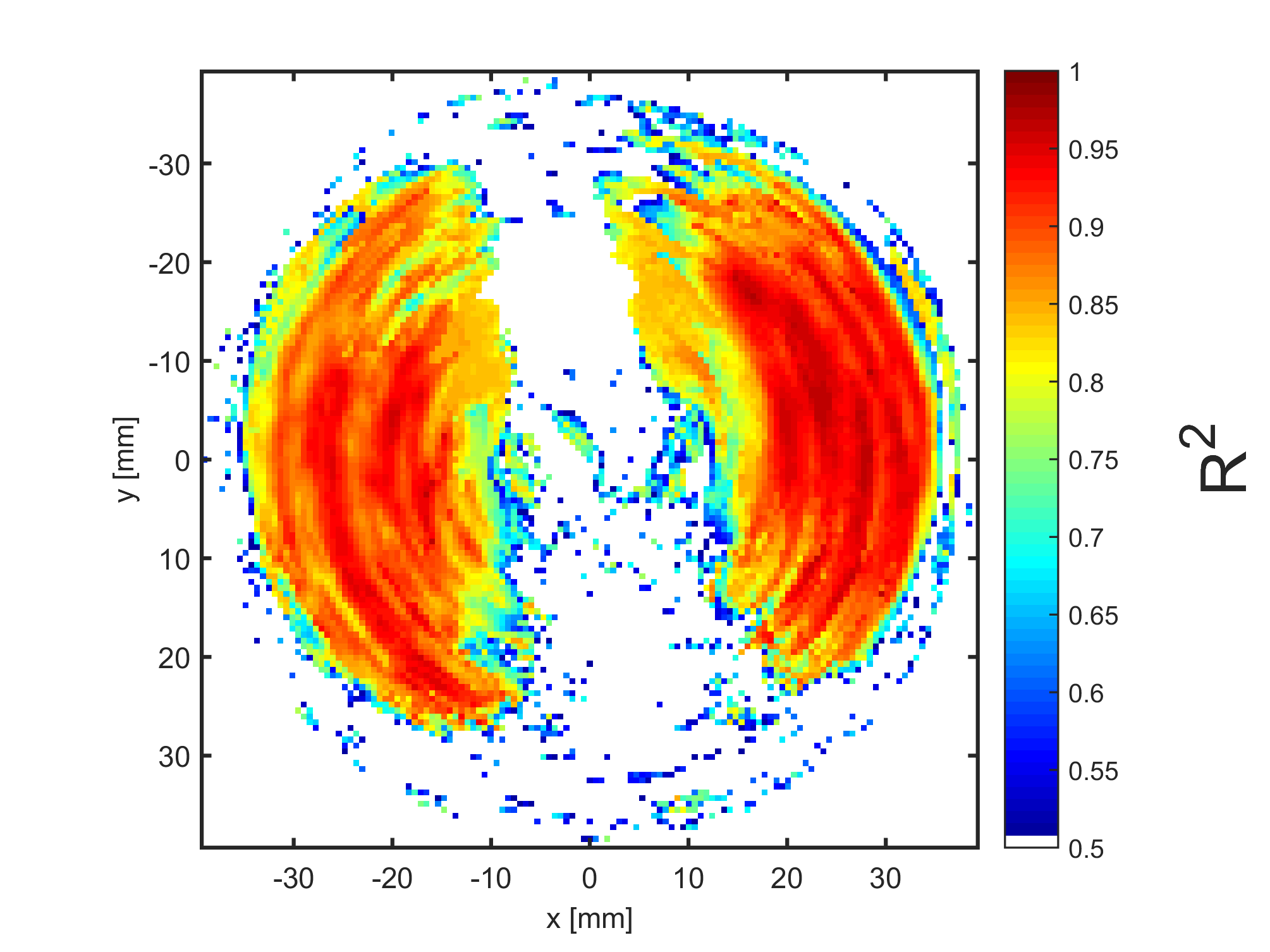

Supplement: Supplementary file 1 [file mmc1.zip › dibData_new/test11_rsquare_dampingMap.png]

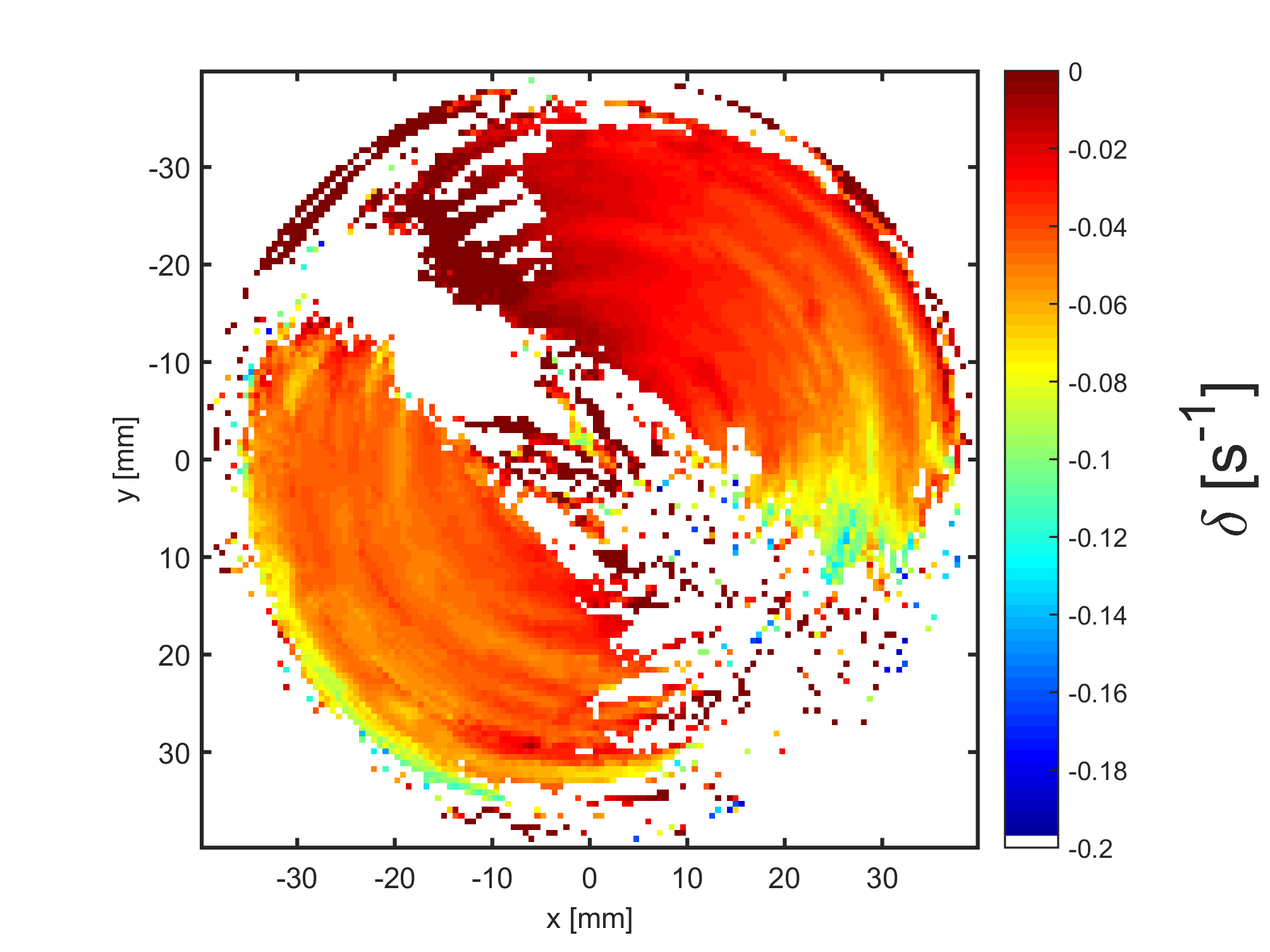

Supplement: Supplementary file 1 [file mmc1.zip › dibData_new/test12_dampingMap.png]

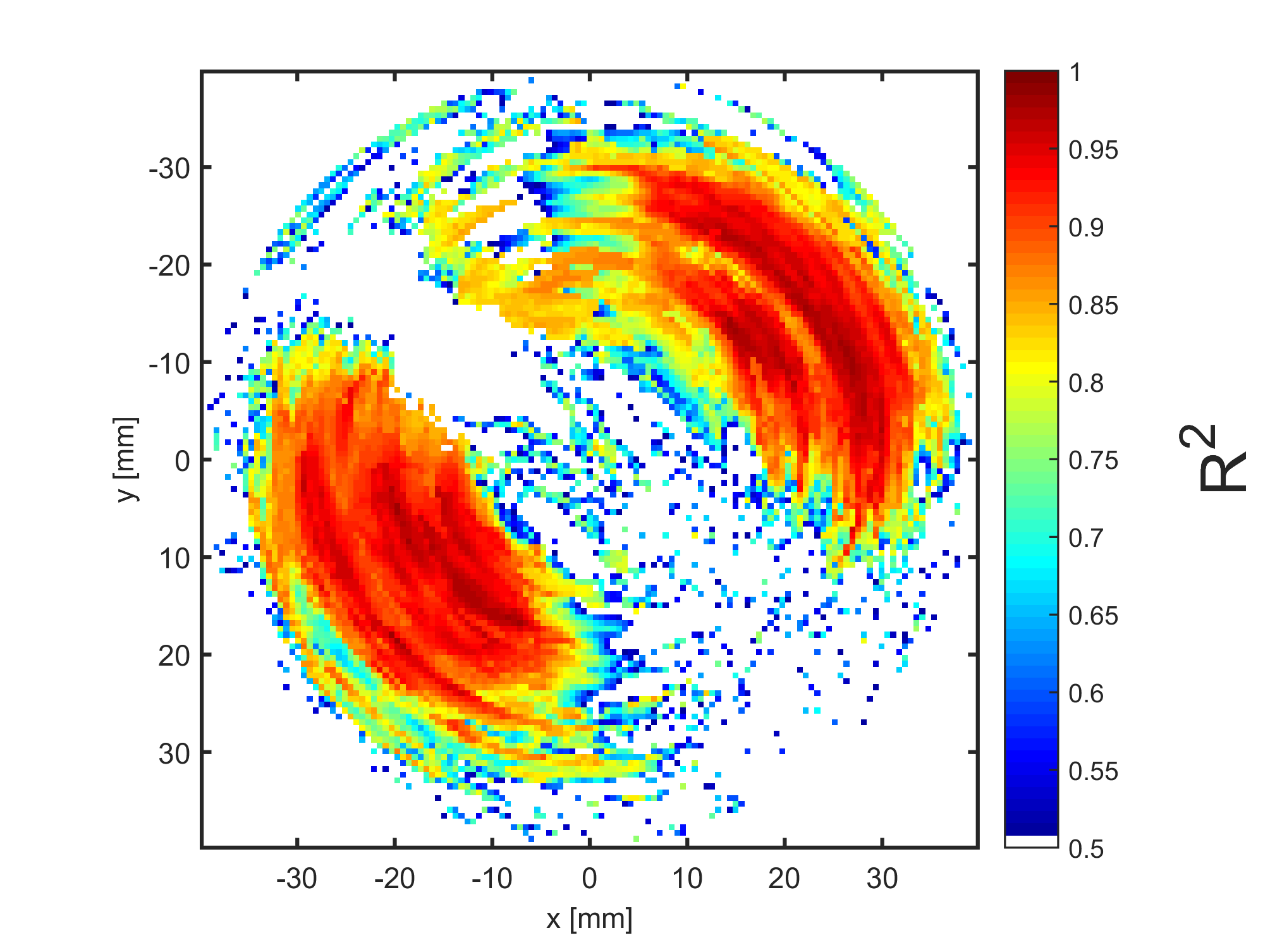

Supplement: Supplementary file 1 [file mmc1.zip › dibData_new/test12_rsquare_dampingMap.png]

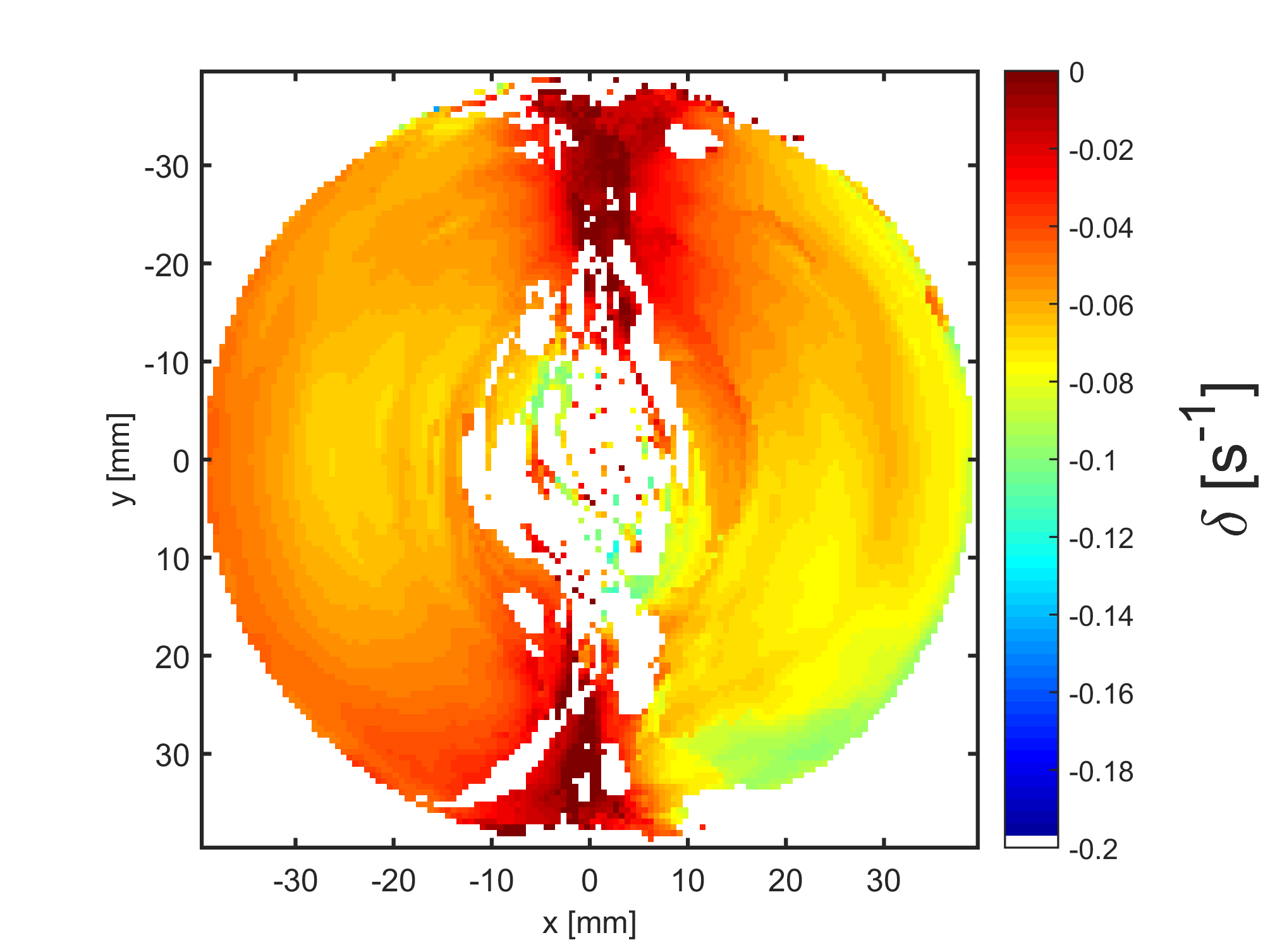

Supplement: Supplementary file 1 [file mmc1.zip › dibData_new/test7_dampingMap.png]

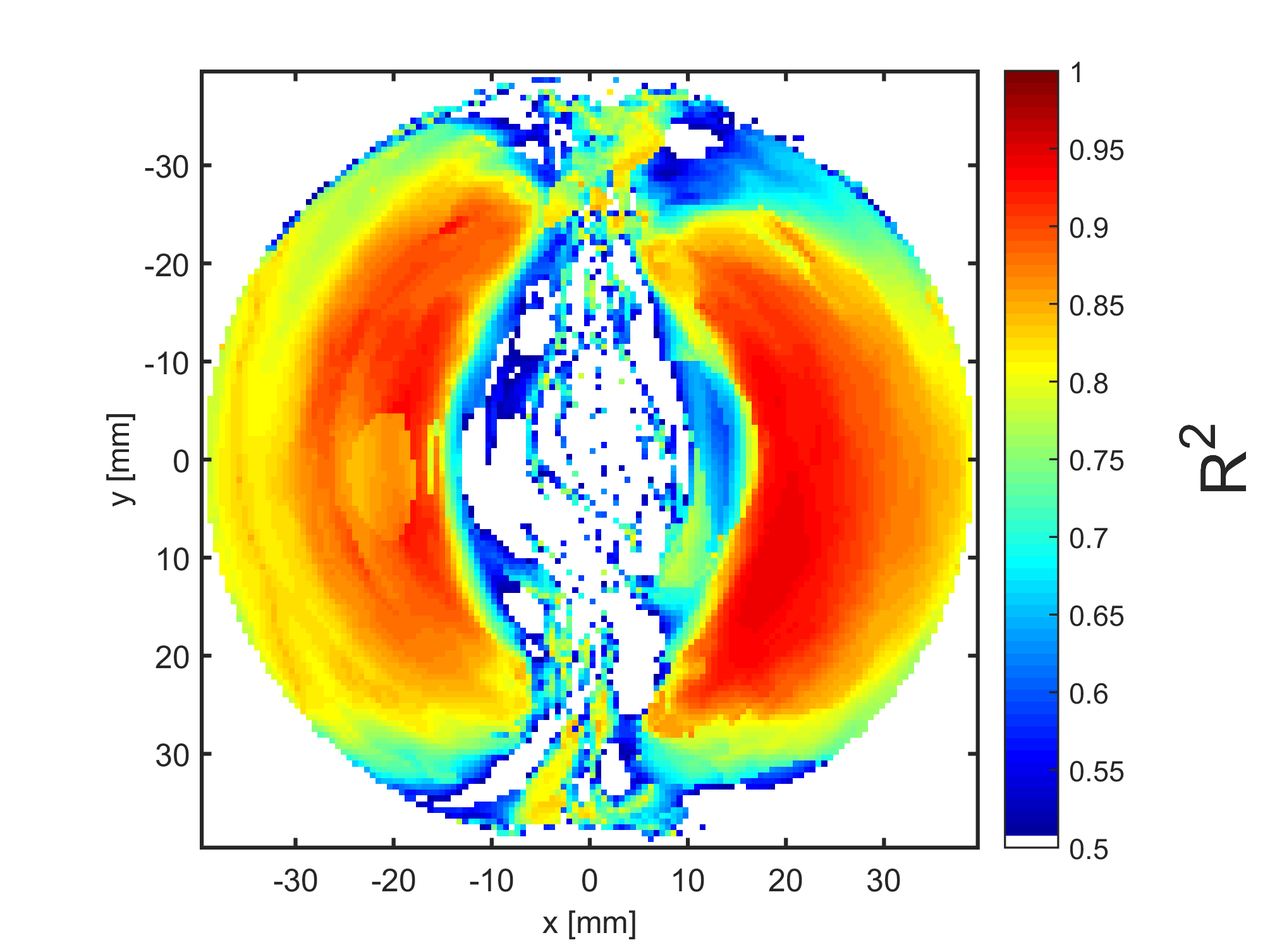

Supplement: Supplementary file 1 [file mmc1.zip › dibData_new/test7_rsquare_dampingMap.png]

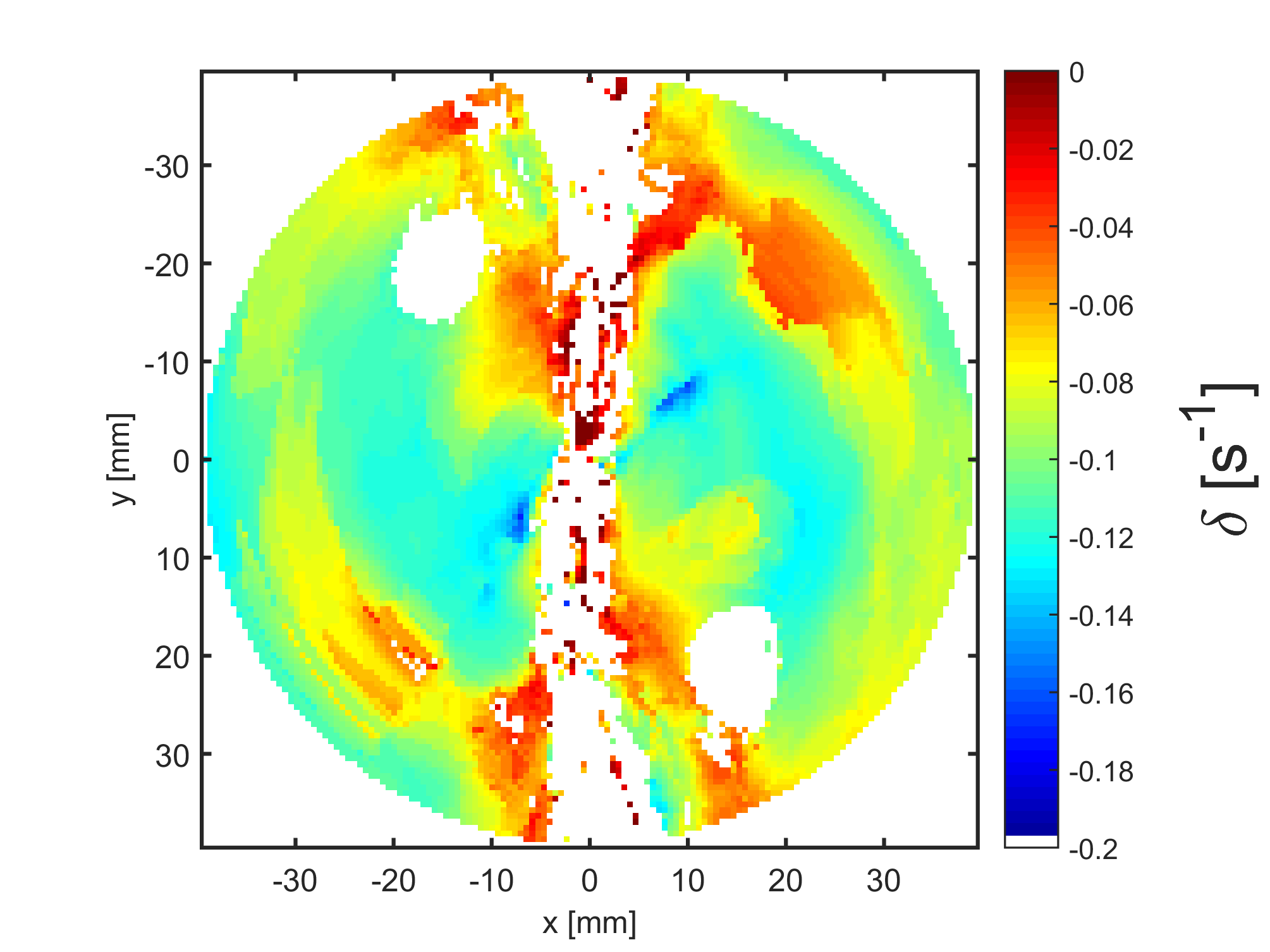

Supplement: Supplementary file 1 [file mmc1.zip › dibData_new/test8_dampingMap.png]

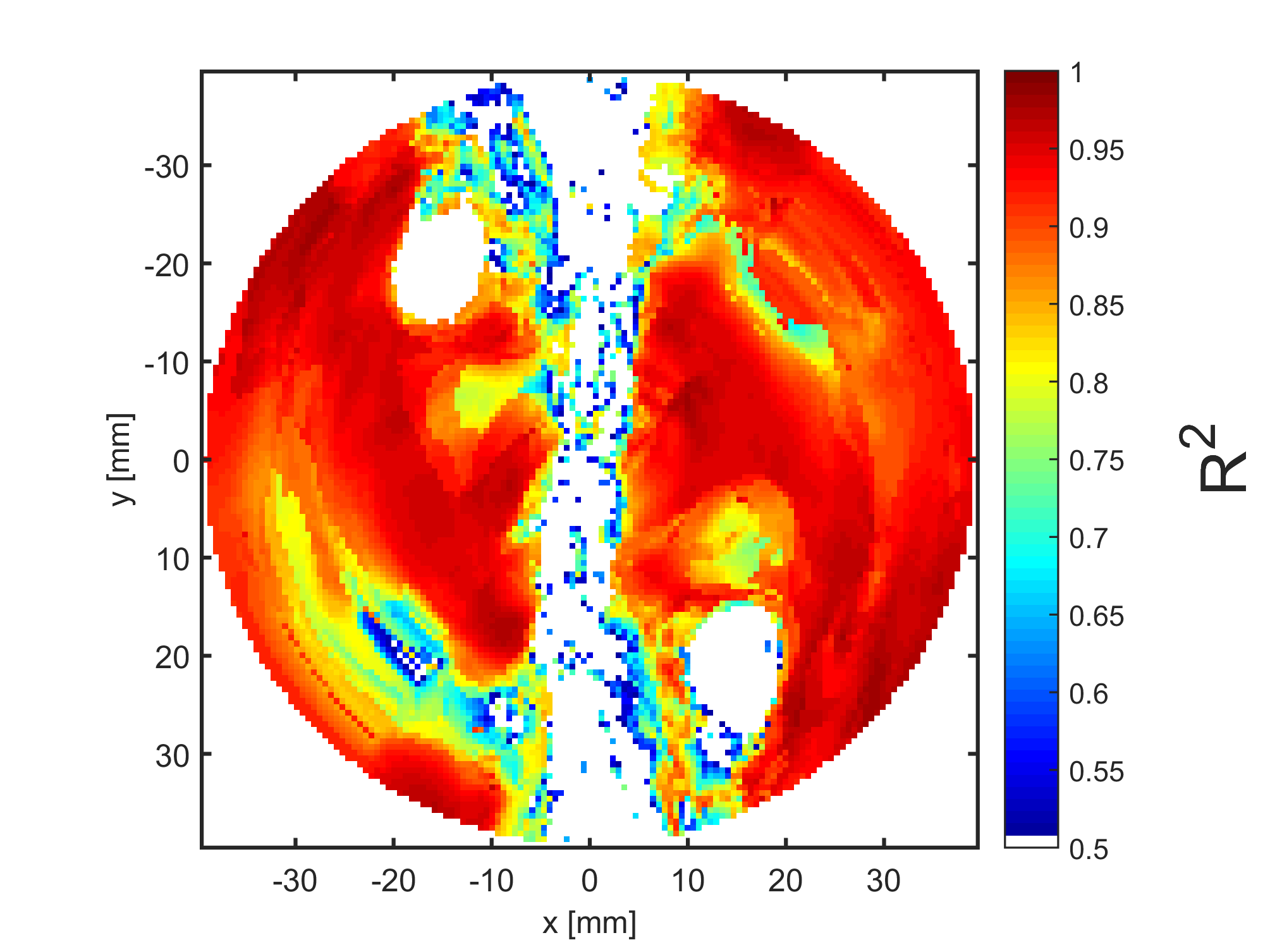

Supplement: Supplementary file 1 [file mmc1.zip › dibData_new/test8_rsquare_dampingMap.png]

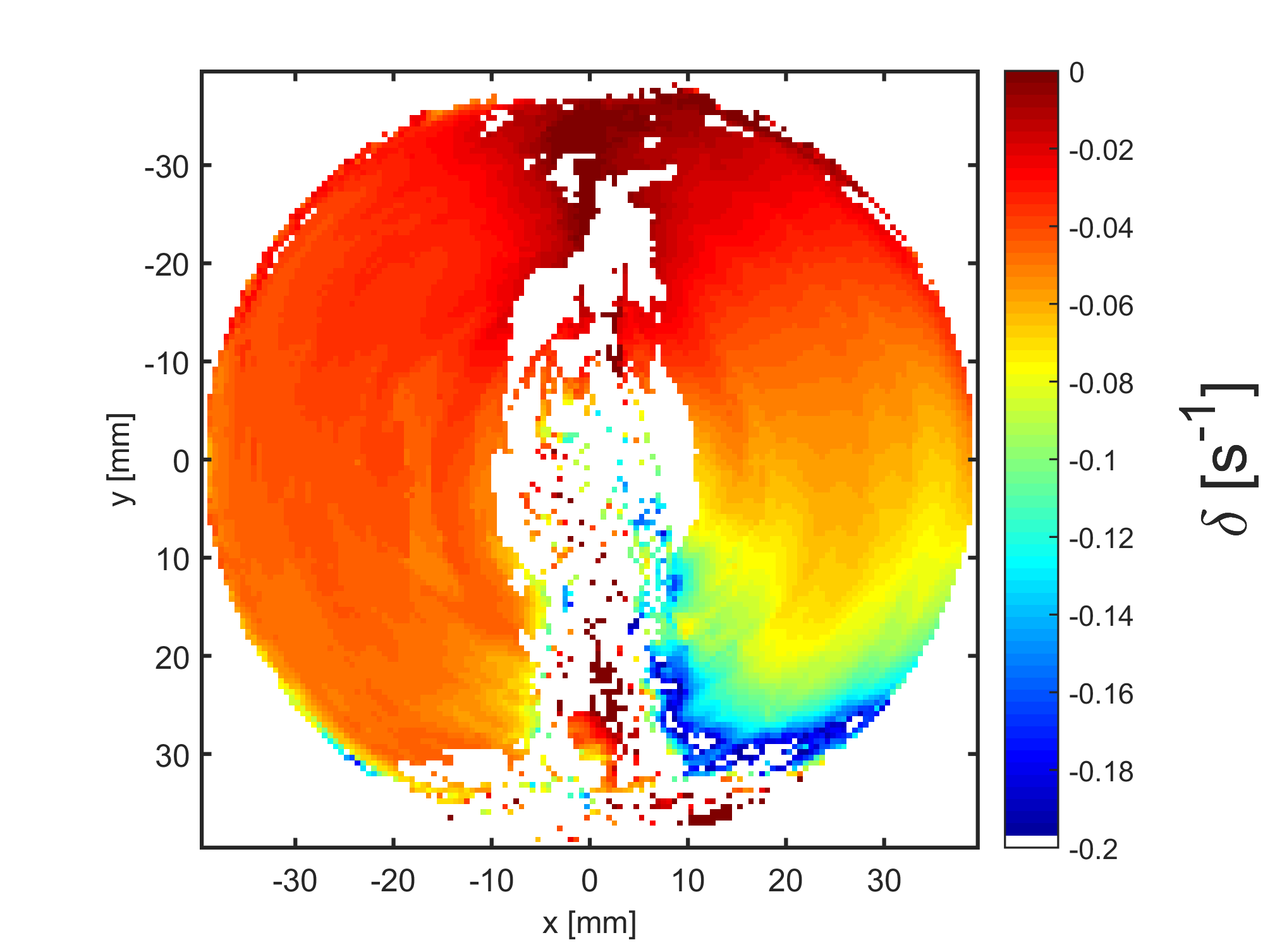

Supplement: Supplementary file 1 [file mmc1.zip › dibData_new/test9_dampingMap.png]

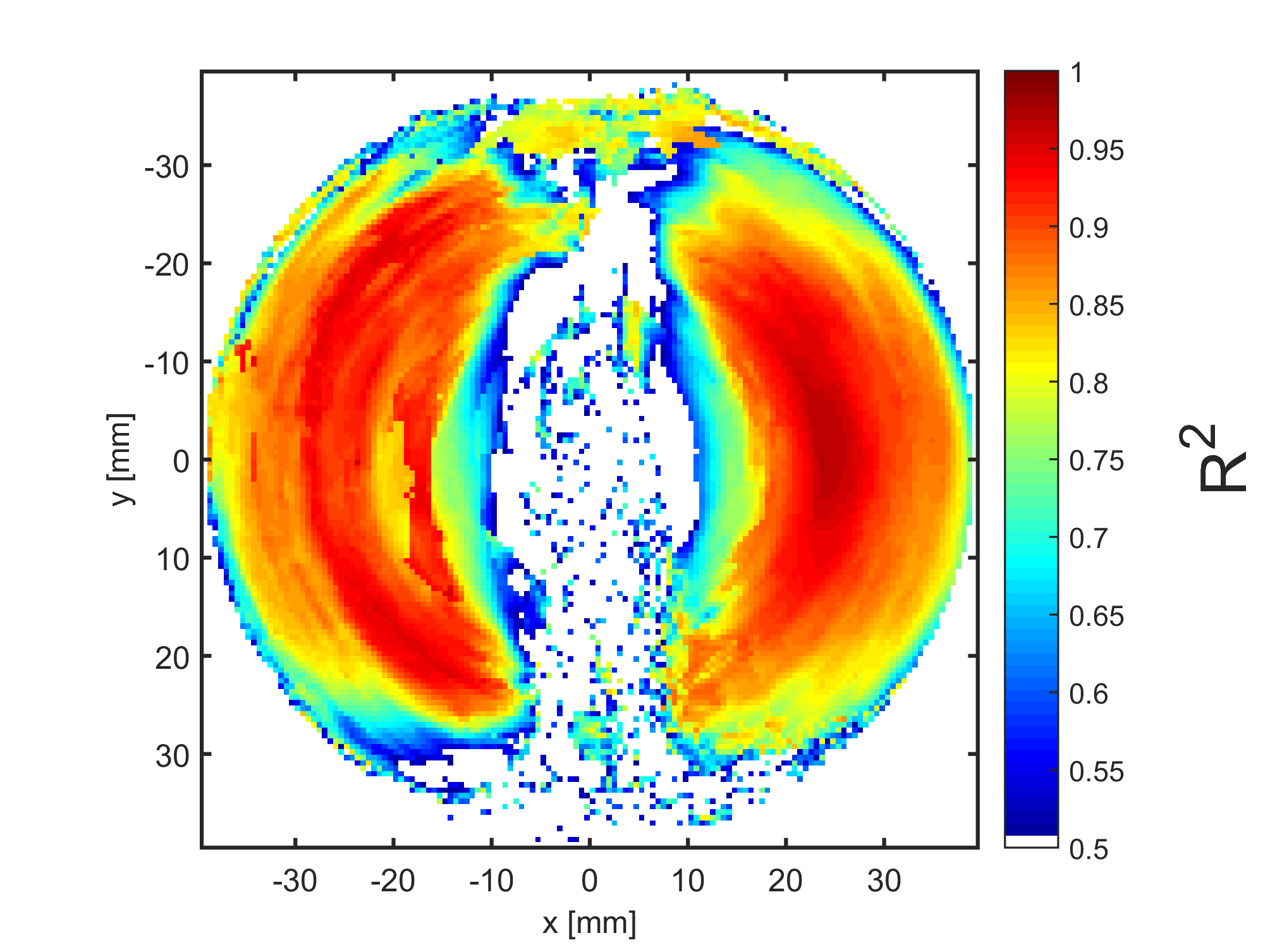

Supplement: Supplementary file 1 [file mmc1.zip › dibData_new/test9_rsquare_dampingMap.png]
